# Les tumeurs des glandes salivaires, étude épidémio-clinique et corrélation anatomoradiologique: étude rétrospective à propos de 148 cas

**DOI:** 10.11604/pamj.2014.19.187.820

**Published:** 2014-10-23

**Authors:** Malika Fassih, Redallah Abada, Sami Rouadi, Mohamed Mahtar, Mohamed Roubal, Mustapha Essaadi, Mohamed Fatmi El Kadiri

**Affiliations:** 1 Service d'ORL et de Chirurgie Cervico-Faciale, Hôpital 20 Août 1953, CHU Ibn Rochd, Casablanca, Maroc

**Keywords:** Tumeurs des glandes salivaires, parotide, tumeur bénigne, tumeur maligne, imagerie, TDM, échographie, IRM, adénome pléomorphe, salivary gland tumors, parotid, benign tumor, malignant tumor, imagery, CT scan, echography, MRI, pleomorphic adenoma

## Abstract

Les tumeurs des glandes salivaires sont rares, elles représentent moins de 3% de l'ensemble des tumeurs. Les tumeurs bénignes sont les plus fréquentes dominées par l'adénome pléomorphe, la glande parotide reste la localisation la plus commune. L'objectif de ce travail est d’évaluer la contribution des 3 méthodes d'imagerie: échographie, TDM et IRM dans la différentiation entre tumeur maligne et lésion bénigne. C'est une étude rétrospective à propos de 148 cas de tumeurs des glandes salivaires collectés sur 5 ans. Les paramètres étudiés étaient l’âge, le sexe du patient, le motif de consultation, les données de l'examen clinique, les données de l'imagerie. Chacun des critères radiologiques utilisés pour déterminer la nature de la tumeur a été analysée et corrélé avec les données de l'histologie. L'analyse s'est basée sur le test du X2 et le calcul du p. Nous avons calculé la sensibilité, la spécificité et l'efficacité diagnostique pour chaque modalité. La localisation parotidienne était prédominante (80%), les tumeurs bénignes ont représenté 76%, dominés par l'adénome pléomorphe. L’échographie a révélé que seulement la présence de quelques critères prédisent le caractère malin de la masse: les limites floues, irrégulières, et la présence d'adénopathies (p < 0,05). A la TDM, seules les limites floues de la masse et l'extension aux tissus adjacents étaient des indicateurs de malignité. A l'IRM, l'irrégularité des contours, l'hyposignal et le signal intermédiaire en séquences T1 et T2, et l'extension aux tissus avoisinants étaient en faveur de la malignité. La corrélation entre résultats de l'imagerie et diagnostic histologique a révélé la supériorité de l'IRM par rapport au scanner et à l’échographie, en termes de sensibilité, spécificité et efficacité diagnostique. L’évaluation préopératoire des tumeurs des glandes salivaires est devenue un challenge pour les ORL et les radiologues, pour prédire la nature de la lésion. L'IRM représente l'examen de choix, notamment avec l'apparition des nouvelles techniques dynamiques.

## Introduction

Les tumeurs des glandes salivaires sont relativement rares, elles représentent moins de 1% de l'ensemble des tumeurs de la tête et du cou. Elles se caractérisent par une grande diversité morphologique et histologique, dominée surtout par les tumeurs bénignes. La connaissance préopératoire de la nature de la tumeur guide le chirurgien dans sa décision chirurgicale; par conséquent, l'imagerie est devenue un véritable outil diagnostique pour les chirurgiens, d'une part, pour préciser le siège exact de la lésion, son extension aux tissus avoisinants, d'autre part, pour prédire la nature maligne ou bénigne de la lésion. L’échographie, couplée ou non à la cytoponction, la TDM et l'IRM, sont les méthodes les plus fréquemment utilisées; cependant, l'efficacité de chacune dans l’évaluation de la nature de la tumeur n'est pas encore bien définie. En effet, dans de nombreuses observations, où l'examen physique suspecte la malignité, les radiologues ne peuvent pas trancher entre tumeur maligne et bénigne; en outre, il existe parfois des discordances entre la réponse des radiologues et le diagnostic histopathologique, ce qui suscite de nombreuses questions: dans quelle mesure l'imagerie peut–elle jouer un rôle dans la différentiation entre tumeur maligne et tumeur bénigne? Quelles sont les caractéristiques radiologiques susceptibles de suggérer la nature maligne de la masse pour chaque méthode? Quelle méthode privilégier par rapport à l'autre en matière de présomption diagnostique? Dans cette étude, nous essayons de répondre à ces questions par une comparaison directe entre les interprétations radiologiques et les diagnostics histologiques.

## Méthodes

C'est une étude rétrospective portant sur 148 patients ayant été suivis et traités pour une tumeur des glandes salivaires: principales et accessoires. La série a été colligée sur une période de 5ans, allant de Avril 2005 jusqu’à Avril 2010, au service d'ORL et chirurgie cervico-faciale à l'hôpital 20 Août de Casablanca. Les paramètres étudiés étaient l’âge et le sexe du patient, le motif et le délai de consultation, l'ensemble des caractéristiques cliniques de la tuméfaction, pouvant suggérer sa nature maligne, étudiées et corrélées aux données de l'histologie. Les données de l'imagerie: l’échographie a été réalisée chez 88 patients, la TDM chez 65, et l'IRM chez 22 malades. Pour chaque méthode diagnostique, nous avons étudié les critères radiologiques utilisés pour distinguer une Tumeur maligne d'une Tumeur Bénigne: Concernant l’échographie, les limites de la masse, ses contours, son aspect, son échogénécité, l'existence d'adénopathies, d'un renforcement postérieur, de zones de nécrose et d'hémorragie, ont été recherchés. A la TDM: nous avons étudié les limites de la masse, ses contours, son comportement à l'injection du produit de contraste, l'extension aux tissus avoisinants, l'existence de nécrose, hémorragies, calcifications, ou de dégénérescence kystique. Pour l'IRM, ont été étudiés, en plus des caractéristiques recherchées à la TDM, le signal de la tumeur en séquences T1W et T2W. Les images radiologiques ont été interprétées par un radiologue spécialisé dans l'imagerie de la tête et du cou. Chacune des images radiologiques a été corrélé à l'histologie, afin de déterminer le caractère prédictif de malignité, l'analyse statistique des résultats s'est basée sur le test de X2 (**chi carré**). Un critère radiologique est significativement indicateur de malignité si le p < 0,05. Le diagnostic proposé par le radiologue (en terme de nature maligne/ bénigne de la tumeur) a été comparé avec le diagnostic histopathologique définitif établit par un anatomopathologiste expérimenté n'ayant aucune information sur le rapport du radiologue. Pour chaque modalité diagnostique, nous avons calculé la sensibilité (définie comme étant la proportion des vrais positifs correctement identifiés par le test), la spécificité (définie comme étant la proportion des vrais négatifs correctement identifiés par le test), et l'efficacité diagnostique ( la proportion des vrais positifs et des vrais négatifs correctement identifiés par le test diagnostique indiquant la concordance entre la diagnostic préopératoire et le résultat histologique définitif).

## Résultats

### Epidémiologie

**Figure 1:**
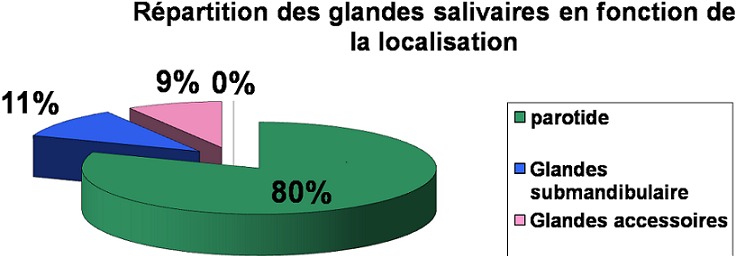
Histologie des tumeurs bénignes

La localisation parotidienne était prédominante (80%), 11% des tumeurs étaient localisées dans la glande submandibulaire. 89% des tumeurs des glandes accessoires étaient situées dans le palais. Aucun cas de tumeur de la glande sublinguale n'a été observé (Fig. 1). L’âge moyen de nos patients était de 51 ans, il était plus élevé pour les tumeurs malignes (61,7 ans) cotre 40 ans pour les tumeurs bénignes. Le pic de fréquence était constaté au niveau de la 4ème décade (50% des malades). Le sexe ratio était de 0,69 avec une légère prédominance féminine (59% des cas).

### Histologie

**Figure 2:**
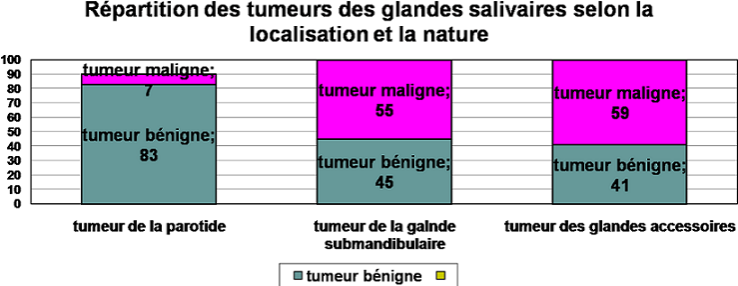
Histologie des tumeurs malignes

Parmi les 148 tumeurs des glandes salivaires, 113 tumeurs étaient bénignes (76%), 35 étaient malignes. Seulement 7% des tumeurs parotidiennes étaient malignes, cette proportion s’élève à 55% dans la glande submandibulaire, et 59% dans les glandes accessoires. Le type histologique le plus fréquent dans le groupe des tumeurs bénignes était l'adénome pléomorphe (75%), suivi du cystadénolymphome (11%) (Figure 1). Pour les tumeurs malignes, le carcinome adénoïde kystique et le carcinome mucoépidérmoïde étaient les types histologiques les plus représentés avec respectivement 38% et 30% (Figure 2).

### Clinique

L’étude clinique des caractéristiques de la tuméfaction et la comparaison avec les résultats de l'histologie en utilisant le test du chi carré, a permis de constater que seulement la présence de quelques symptômes prédisent le caractère malin de la masse: la paralysie faciale, la fixité aux plans adjacents, les limites mal définies, et la douleur sont des indicateurs statistiquement significatifs de malignité (p < 0,01) (Tableau 1).

**Tableau 1:** Caractéristiques cliniques des tumeurs bénignes et malignes des glandes salivaires

Caractéristiques de la tuméfaction	Tumeur bénigne N = 113	Tumeur maligne n= 35	Valeur du p (x2-test)
**Consistance**			
dure	36	15	< 0,30
ferme	68	20	
molle	9	-	
**Limites**			
nette	73	13	< 0,01
mal définie	40	22	
Mobile	76	11	< 0,001
Fixe / 2 plans	37	24	
Douleur	33	34	< 0,001
Paralysie faciale	0	14	< 0,001
Coliques salivaires (obstruction Wharton)	0	1	< 0,9
Adénopathies	40	17	< 0,10

### Imagerie

Chacun des critères radiologiques utilisés pour distinguer une Tumeur maligne d'une Tumeur bénigne a été étudié et comparé avec les données de l'histologie. Les résultats ont été analysés par le X2 test. Concernant l’échographie, réalisée chez 88 malades (59%), les limites floues, irrégulières, la présence d'ADP sont des indicateurs statistiquement significatifs de malignité (p < 0,01). Le caractère hypoéchogène, les limites nettes, les contours réguliers ou lobulés sont des indicateurs statistiquement significatifs de bénignité. La présence de nécrose, d'hémorragie, l'aspect hétérogène, et le renforcement postérieur sont des critères non significatifs (Tableau 2). La TDM a été réalisée chez 65 malades (44%). Parmi les caractéristiques étudiées, seules les limites floues de la masse, et l'extension aux tissus adjacents étaient des indicateurs statistiquement significatifs pour prédire la malignité (< 0,001). La nécrose, la dégénérescence kystique, les calcifications, et la prise de contraste n'avaient pas de valeur significative (Tableau 3). L'IRM a été demandée chez seulement 22 malades (15%). L'irrégularité des contours, l'hyposignal et le signal intermédiaire en séquence T1 et T2, et l'extension aux tissus avoisinants étaient statistiquement en faveur de la malignité (< 0,01) (Tableau 4).

**Tableau 2:** Résultats des échographiques dans les tumeurs bénignes et les tumeurs malignes des glandes salivaires (n = 88)

Caractéristiques échographiques	Tumeurs bénignes n= 80	Tumeurs malignes n= 8	Valeur du *P* (x2-test)
**Limites**			
nettes	80	2	< 0,001
floues	0	6	
**Contours**			
réguliers	44	2	< 0,001
lobulés	36	0	
irréguliers	0	6	
**Aspect**			
homogène	38	2	< 0,90
hétérogène	42	6	
**Echogénécité**			
hyper	5	2	< 0,001
Iso	0	3	
hypo	75	3	
Zone de Nécrose / hémorragies	36	6	< 0,20
Adénopathies	41	8	< 0,01
Renforcement postérieur	38	6	< 0,90

**Tableau 3:** Résultats des radiologiques en TDM dans les tumeurs bénignes et les tumeurs malignes (n = 65)

Caractéristiques radiologiques	Tumeur bénigne n = 30	Tumeur maligne n = 35	valeur du *P* (x2-test)
**Limites**			
nettes	28	12	< 0,001
flous	2	23	
**contours**			
régulier	30	15	< 0,50
irrégulier	0	13	
Extension aux structures adjacentes	3	22	< 0,001
Réhaussement au produit de contrast	26	32	< 0,90
Nécrose /hémorragie	10	17	< 0,30
Dégénérescence kystiques	7	8	< 0,90
calcifications	5	4	<0,90

**Tableau 4:** Critères radiologiques en IRM dans la distinction entre tumeurs bénignes et tumeurs malignes (n = 22)

Caractéristiques en IRM	Tumeurs bénignes n = 2	Tumeurs malignes n = 20	Valeur du *P* (x2-test)
**Contours**			
régulier	2	0	< 0,01
lobulé	-	2	
irrégulier	-	18	
Extension aux structures adjacentes	0	16	< 0,001
**Signal (T1):**			
hyper	-	-	< 0,001
intermédiaire			
2			
12		
hypo	2	12	
**Signal (T2)**:			
hyper	2	1	< 0,001
intermédiaire	-	8	
hypo	-	11	
Rehaussement au Produit de contraste	2	10	< 0,10
Nécrose /hémorragie	-	6	< 0,5
Dégénérescence kystiques	-	5	< 0,5

Se basant sur ces résultats, cette étude a essayé de déterminer l'efficacité de chaque méthode d'imagerie dans la différenciation entre tumeur maligne et tumeur bénigne. Nous avons entrepris une corrélation entre l'interprétation des radiologues et le diagnostic histopathologique définitif, en terme de détection de la nature de la tumeur (maligne/ bénigne). En effet, 72 images échographiques étaient interprétées comme bénignes par les radiologues, dont 2 était malignes à l'histologie définitive, c’était un carcinome mucoépidérmoide dans les 2 cas. Par ailleurs, parmi les 16 diagnostics en faveur de la malignité, suggérés par les radiologues, 10 tumeurs (62%) étaient bénignes, l'adénome pléomorphe a été retrouvé dans 8 cas. La discordance entre l’échographie et l'histologie était observée dans 13,6%. Le diagnostic proposé par l’échographie était correct pour la détection de malignité dans 75%. La spécificité était de 87,5%.

Concernant la TDM, le diagnostic radiologique était correct dans la détection de malignité dans 71,5%, la discordance entre la TDM et l'histologie a été noté dans 23% des cas: 10 (28%) des tumeurs malignes ont été faussement interprétées comme tumeurs bénignes par le scanner, il s'agissait de 7 carcinomes mucoépidermoïdes, 2 carcinomes adénoïdes kystiques et 1 lymphome. D'autre part, 5 parmi 30 patients (16%) avaient une tumeur histologiquement bénigne alors que l'interprétation du scanner était en faveur de la malignité. L'adénome pléomorphe était le plus représenté (4 cas). La spécificité était de 83%. Le diagnostic proposé par l'IRM était correct dans 100% des cas. La spécificité était de 100%. L'efficacité diagnostique était de 86,4% pour l’échographie, 77% pour la TDM, et atteint 100% avec l'IRM.

## Discussion

Les Tumeurs des glandes salivaires représentent moins de 3% de toutes les tumeurs [1]. La plupart d′entre elles sont bénignes. En règle générale, le risque de malignité est inversement proportionnel à la taille de la glande où se développe la tumeur. Le pourcentage de tumeurs malignes dans la glande parotide est de 20-30%, il atteint 45-60% dans la glande sub-mandibulaire et 70-85% dans la glande sublinguale, il est de 49-80% dans les glandes salivaires accessoires [2, 3]. La malignité des tumeurs des glandes salivaires peut être suspectée en se basant sur un faisceau d'arguments cliniques et paracliniques. Sur le plan clinique, la douleur, la paralysie faciale et l'atteinte ganglionnaire doivent attirer l'attention du clinicien [4]. Selon Jouzdani [5], certains signes cliniques ont été associés à la malignité: masse dure (48%), paralysie faciale (21%), adénomégalie (11,5%), invasion cutanée (5,5%). Pour Ahuja [6], la douleur est retrouvée chez 5,1% des malades avec une tumeur bénigne, et 6,5% des patients suivis pour tumeurs malignes; par conséquent, la douleur ne peut pas être un bon indicateur pour suspecter la malignité. L'augmentation rapide de taille oriente plutôt vers les lymphomes, les carcinomes épidermoides et les tumeurs indifférenciées. Dans notre série, une tumeur maligne était suspectée devant une masse irrégulière et fixe par rapport aux plans adjacents; s'associant à une parésie faciale (en cas de tumeur parotidienne) et des douleurs, seulement ces critères étaient des indicateurs statistiquement significatifs de malignité. L'adénome pléomorphe, représentant le contingent prédominant des tumeurs bénignes, a été suspecté devant une tuméfaction grossièrement arrondie, bien limitée, de consistance ferme, qui a augmenté progressivement de taille. Toutefois, leur valeur diagnostique n'est pas absolue. Des auteurs rapportent 9% de tumeurs malignes évoluant depuis plus de 10 ans [7, 8]. Dans notre série, le délai de consultation maximal pour les tumeurs malignes était deux ans.

Sur le plan paraclinique, le rôle de l'imagerie dans l’évaluation des tumeurs des glandes salivaires est de définir la localisation intra ou extra-glandulaire, évaluer l'extension locale et l'invasion des tissus avoisinants, détecter les caractéristiques orientant vers la malignité, et les métastases ganglionnaires. La sensibilité de l’échographie dans la détection des tumeurs du lobe superficiel de la parotide est voisine de 100% [9]. Concernant la distinction entre lésion glandulaire et extra glandulaire, l'efficacité diagnostique de l’échographie est de 95%, et de 100% selon Fontanel [10]. L’échographie permet d'orienter vers la malignité dans 80% des cas [9]. Dans notre étude, les critères sur lesquels se sont basés les radiologues pour prédire la malignité, étaient les limites floues, irrégulières, et la présence d'ADP. Pour Burke [11], c'est le caractère mal défini, hypoéchogène et hétérogène de la masse, avec un renforcement postérieur, qui suggèrent la malignité. Pour Bradley [12], la tumeur maligne apparait mal définie, d'architecture hétérogène, avec nécrose interne et dégénérescence kystique. Au doppler, la masse est hypervascularisée. Notre étude a démontré que l’échographie n'est nullement inférieure à la TDM dans l’évaluation de la nature de la tumeur; à l'inverse, la capacité de l’échographie à détecter les tumeurs malignes était légèrement supérieure à celle de la TDM: sensibilité à 75% pour l’échographie versus 71,5% pour la TDM, mais sans différence significative. Pour Rudack [13], l’échographie était comparable aussi bien à la TDM qu’à l'IRM en matière de distinction entre tumeur maligne et bénigne, il n'y avait pas de différence statistiquement significative entre la sensibilité et la spécificité de chacune des modalités diagnostiques. Ces résultats sont expliqués en partie par le fait que la plupart des tumeurs sont aux dépens du lobe superficiel de la parotide et de la glande submandibulaire, qui sont très bien explorés par l’échographie. Gritzmann [14] a démontré dans son étude rétrospective de 287 cas, que l’échographie peut analyser les tumeurs superficielles de la parotide et de la glande sub mandibulaire avec la même précision que la TDM et l'IRM. Le même résultat a été constaté par Burke [11], l’échographie a pu correctement faire la différence entre tumeur maligne et bénigne dans 90% des cas. Concernant la distinction entre lésion glandulaire et extra glandulaire, l'efficacité diagnostique de l’échographie était de 95%. Les limites de l’échographie sont représentées par les tumeurs de dimensions importantes, les tumeurs au dépens du lobe profond, du prolongement parapharyngé et des glandes accessoires, l’étude de l'extension osseuse et notamment à la base du crane [15, 16]. Aussi, la détection des adénopathies rétro-pharyngées, premier relai de la muqueuse de la cavité orale et pharyngée, échappe à l’échographie. La TDM complète l'exploration du lobe profond de la parotide et précise la topographie des lésions et leur extension locorégionale. Elle atteste de l'agressivité de certaines tumeurs malignes et l'envahissement des tissus de voisinage. Toutefois, il n'existe pas de critère tomodensitométrique spécifique de la nature tumorale [10, 17]. Selon Akkari [4], l'aspect hétérogène de la masse, ses limites irrégulières, le rehaussement massif à l'injection du produit de contraste et la présence d'adénopathies satellites étaient en faveur de la nature maligne. Cependant la valeur diagnostique de l'IRM reste meilleure [18, 19]. Selon Devos [20], les performances diagnostiques de l'IRM par rapport à l’étude histologique étaient les suivantes: sensibilité = 79%, spécificité = 100%. Une discordance entre les résultats de l'imagerie et ceux de l’étude anatomopathologique n'a concerné que 4,3% des cas; dans notre série, le rapport de l'IRM était concordant avec les résultats anatomopathologique dans 100% des cas en termes de différenciation entre tumeur bénigne et maligne. Cette étude a montré la nette supériorité de l'IRM en termes de différenciation entre tumeur bénigne et maligne par rapport au scanner et l’échographie. Le diagnostic apporté par l'IRM était correct dans tous les cas où elle a été demandée. Notre constat s'accorde avec plusieurs études [21, 22] qui ont prouvé que l'IRM représente la méthode de choix pour l'exploration des tumeurs des glandes salivaires. Outre sa capacité de déterminer le siège exact intra ou extra-glandulaire de la tumeur, d’étudier le lobe profond de la parotide et l'extension tumorale précise en profondeur vers les tissus adjacents et l'invasion périneurale, elle offre une meilleure approche des lésions grâce aux coupes coronales et sagittales qu'elle procure, qui représente un avantage par rapport à la TDM [23]. Par ailleurs, K.H.Kim [23] a démontré dans son étude que la TDM était comparable à l'IRM dans l’évaluation de la nature tumorale, la sensibilité et la spécificité étaient respectivement de 93% et 61% pour la TDM, et de 83% et 63% pour l'IRM. Koyuncu [24] a démontré que les deux modalités diagnostiques procurent les mêmes informations en matière d’évaluation préopératoire, les images radiologiques indiquant la malignité sont presque les mêmes pour la TDM et l'IRM: les limites floues, l'irrégularité des contours, l'extension aux tissus adjacents. Cependant, l'IRM apporte un critère supplémentaire, qui est l'intensité de la lésion en T1 et T2. Joe and Westesson [25] ont indiqué que les carcinomes paraissent en hyposignal et en signal intermédiaire. D'autres séries ont démontré que seuls les carcinomes de haut grade obéissent à cette règle, les carcinomes de bas grade paraissent en hypersignal en T2 simulant une tumeur bénigne [16]. Dans notre étude, 19 parmi les 20 tumeurs malignes diagnostiquées en IRM, étaient en hypo signal ou en signal intermédiaire en T1 et en T2, ce critère était statistiquement en faveur de la malignité.

Se rapportant aux résultats de cette étude, exceptée l'IRM dont la proportion d'erreur était nulle, il est difficile de se fier aux seuls résultats de l’échographie et de la TDM dans la différenciation entre tumeur maligne et bénigne. Les deux modalités diagnostiques présentent un taux d'erreur diagnostique non négligeable. Le pourcentage d'erreur dans l'interprétation de tumeurs malignes comme étant bénignes était plus élevé pour le scanner (28%), par rapport à l’échographie (3%). Ce qui nous incite à choisir l’échographie comme examen de première intention pour l’étude des tumeurs des glandes salivaires, la même constatation a été rapportée par Burke [11]. Rudak [13] considère l’échographie performante dans le diagnostic des tumeurs bénignes. En effet, l’échographie a établi le diagnostic dans 54% des tumeurs bénignes. Cependant, dans notre étude, l’échographie avait une nette tendance à l'erreur concernant l'interprétation de tumeurs bénignes comme étant malignes (62%) par rapport au scanner (16%). Pour Kim [23], aussi bien la TDM et l'IRM avaient une tendance élevée à l'erreur surtout pour l'interprétation de tumeurs bénignes comme tumeurs malignes: le taux d'erreur était de 39% pour la TDM et 35% pour l'IRM. Par conséquent, il suggère que le scanner devrait être l'examen de première intention dans l’évaluation initiale des tumeurs des glandes salivaires. Pour Rudak, le diagnostic suggéré par les radiologues concernant les tumeurs malignes était correct dans des proportions faibles pour les 3 modalités: 4 cas parmi 30 pour l’échographie, 7cas de 23 pour l'IRM, et 1 cas parmi 8 pour la TDM. Dans notre série, l'IRM était performante et supplantait le scanner et l’échographie aussi bien pour le diagnostic des tumeurs bénignes ou malignes. Cette différence de résultats, entre notre étude et celles de Rudak [13] et Kim [23] est probablement due au nombre limité de malades ayant bénéficié d'IRM dans notre série, ce qui a réduit considérablement la marge d'erreur (22/ 148 comparativement avec la série de Rudak: 109/582, et Kim 31/147. Au terme de ce travail, on peut déduire que l’échographie devrait être l'examen initial à réaliser devant la palpation d'une masse aux dépens de glandes salivaires. L'IRM trouvera son indication lorsque les renseignements apportés par l’échographie sont insuffisants pour le chirurgien.

## Conclusion

L’évaluation précise préopératoire des tumeurs des glandes salivaires est devenue un véritable challenge pour les ORL et radiologues, pour préciser le siège exact de la masse, l'extension précise aux tissus avoisinants, pour prédire la nature de la lésion et guider ainsi la décision thérapeutique. La distinction entre tumeur maligne et tumeur bénigne est parfois difficile surtout pour les tumeurs malignes de bas grade qui sont difficiles à différencier des tumeurs bénignes; mais aussi pour les adénomes pléomorphes de grandes tailles qui sont difficilement distingués des tumeurs malignes. D'où la nécessité de développer et généraliser les nouvelles techniques dynamiques de l'IRM telles que la mesure du coefficient de diffusion, et -MR spectroscopy- qui ont démontré des résultats prometteurs en matière de différenciation entre tumeur maligne et tumeur bénigne, et même dans le diagnostic étiologique: différencier entre adénome pléomorphe et tumeur de Warthin, et entre ces deux entités et les tumeurs malignes.
